# RNA Replicons - A New Approach for Influenza Virus Immunoprophylaxis

**DOI:** 10.3390/v2020413

**Published:** 2010-01-29

**Authors:** Gert Zimmer

**Affiliations:** Institute of Virology and Immunoprophylaxis (IVI), Sensemattstrasse 293, CH-3147 Mittelhäusern, Switzerland; E-Mail: gert.zimmer@ivi.admin.ch; Tel.: +41-31-848-9211; Fax: +41-31-848-9222

**Keywords:** RNA replicon, vaccine, biosafety, cellular immunity, mucosal immunity, heterosubtypic immunity, alphavirus, vesicular stomatitis virus

## Abstract

RNA replicons are derived from either positive- or negative-strand RNA viruses. They represent disabled virus vectors that are not only avirulent, but also unable to revert to virulence. Due to autonomous RNA replication, RNA replicons are able to drive high level, cytosolic expression of recombinant antigens stimulating both the humoral and the cellular branch of the immune system. This review provides an update on the available literature covering influenza virus vaccines based on RNA replicons. The pros and cons of these vaccine strategies will be discussed and future perspectives disclosed.

## 1. Introduction

Influenza viruses are highly contagious, rapidly evolving respiratory pathogens with established lineages in avian and mammalian species. Human influenza viruses circulate worldwide, causing annual epidemics associated with three to five million cases of severe illness and 250,000 to 500,000 deaths (www.who.int/mediacentre/factsheets/fs211/en). In temperate regions, annual influenza epidemics usually start in November and last until March. Influenza viruses can affect persons at any age, but the elderly (>65 years), young children (<2 years) and persons with underlying chronic diseases or weakened immune systems are particularly susceptible to severe and sometimes fatal respiratory distress symptoms [1].

In addition to the recurrent annual epidemics, influenza A viruses may cause worldwide pandemics from time to time. Pandemic influenza viruses of the past could be related to avian influenza viruses (AIV) that had either adapted to infect human cells or had generated reassortants with mammalian influenza viruses. Pandemic viruses usually show major antigenic differences from the current circulating human influenza viruses (“antigenic shift”). Accordingly, they will encounter a human population with little pre-existing immunity, allowing for rapid global virus spread. The epidemic of highly pathogenic AIV H5N1 which started 2003 in Southeast Asia and spread to Europe and Africa exemplifies the zoonotic potential of AIV [4]. Fortunately, the susceptibility of humans to this virus was relatively low and transmission from humans to humans did not occur. However, the situation may change if H5N1 adapts to humans or generates reassortants with mammalian influenza viruses, such viruses presenting a potential new pandemic. Certainly, this was the case in 2009 when a new pandemic influenza virus of subtype H1N1 emerged. This virus arising from reassortants which themselves had originated from avian, swine and human ancestors showed clearly the high level of threat and rapidity of global spread when a new influenza virus was successfully introduced into the human population.

### Potential obstacles with current influenza vaccines

Current human influenza virus vaccines rely on inactivated split vaccines containing the major viral surface antigens HA and NA. The existing seasonal vaccines employ antigens of the most prevalent human influenza viruses, namely influenza A virus subtypes H3N2 and H1N1 together with an influenza B virus strain, present as a triple vaccine for parenteral application. According to WHO recommendations, the vaccine composition is updated every year to match the rapid “antigenic drift” of the viruses. However, vaccine efficacy is strongly influenced by the age and immunocompetence of the recipient, as well as the degree of similarity between the vaccine strains and the viruses circulating during an influenza season [2,3].

The annual production cycle for conventional influenza vaccines must be initiated at least 6 months before the vaccine can become available [7]. It begins with the selection of “target” strains that are believed to be the best match for inducing effective immunity against those viruses likely to pose a health threat in the upcoming flu season. Using these strains, the vaccine manufacturers start to prepare reassortants with fast growing high-yield donor strains. Following propagation of these “seed” strains in embryonated chicken eggs, the viruses are inactivated and partially purified for preparing the core vaccine material. With present-day influenza virus vaccines, the vaccine material is “split” prior to formulation and testing for immunogenicity and safety. The final influenza virus subunit vaccine contains the envelope glycoproteins HA and NA but lacks other influenza virus antigens as well as the viral RNA.

Generally, the current split vaccines for influenza induce good serum antibody responses, but are less efficient in triggering mucosal IgA antibodies. Vaccination is most often done by the parenteral route, which is not ideal for inducing mucosal immunity. In addition, these split vaccines may be poor at eliciting the cellular immune responses which are probably important in the context of cross-protectivity [8,9]. Thus, inactivated split vaccines are considered to be effective primarily in the sense of the HA they contain. They will only be sufficiently effective if the major antigen HA of the presently circulating strain closely matches the antigenic structure of the HA of the seed strain selected 6 months before [10].

Pandemic influenza virus vaccines encounter additional obstacles. They often have to be supplied at a particularly short notice in sufficiently large quantities to cover the immunization of several millions of people. However, the limited availability of specific pathogen-free embryonated chicken eggs may hinder meeting this demand, and can even prove insufficient for production of the required vaccine doses to control large seasonal influenza epidemics. Moreover, one cannot guarantee that all influenza viruses will be equally efficient at producing the required amounts of antigen. For example, HA of certain potential pandemic H5N1 strains were found to be less immunogenic, making high antigen doses necessary to achieve appreciable antibody responses [11]. Current strategies to solve these problems include vaccine production on certified mammalian cell lines and use of novel adjuvants that allow antigen sparing [11]. Nevertheless, whatever system is used for propagating pandemic influenza viruses, biosafety containment levels are required (at least BSL3 for the original virus and at least BSL2+ for any reassorted or reverse genetics-engineered vaccine virus), thus further complicating the production of pandemic vaccines.

Another approach taken for influenza virus vaccine production was that of cold-adapted influenza virus vaccines, extensively employed in Russia in the past [12]. A cold-adapted virus (FluMist) was also licensed in the US as an epidemic human influenza vaccine [13]. Compared to inactivated vaccines, live attenuated influenza vaccines (LAIV) have the advantage to induce broader and longer-lasting immune responses, including cellular and mucosal immunity. Indeed, LAIV have demonstrated higher levels of heterosubtypic protection [14]. While this turned out to be true for seasonal LAIV bearing human H1, H3 or B HA antigens, LAIV bearing avian H5 HA antigens showed highly restricted replication in humans resulting in very low antibody responses [15]. It is also important to note that cold-adapted influenza virus vaccines have been approved in the US only for healthy children and adults between two and 49 years of age. The vaccine must not be applied to immunocompromised persons and to adults 50 years or older [16]. In addition, it cannot be ruled out that LAIV regain virulence by either mutational reversion or reassortment with field viruses.

None of the classical vaccine concepts (inactivated or live attenuated viruses) perfectly satisfies the demands for seasonal and pandemic influenza virus vaccines. Ideally, a high-quality influenza virus vaccine would integrate both the safety of inactivated vaccines and the efficacy of live attenuated vaccines. An ideal vaccine is expected to provide long-lasting and heterosubtypic protection, and should be available at short notice in sufficient quantities to face an influenza pandemic. The recent race to produce adequate doses of vaccine against the new H1N1 2009 virus exemplifies the critical nature of this problem. While the virus was continuing to induce disease during the spring and summer months of 2009, vaccines were not available before November 2009. In the future, it is important to identify means of producing influenza virus vaccines more rapidly, and increasing the potential for producing efficacious vaccines with the capacity of inducing both humoral and cellular immunity. In this context, RNA replicons offer major advantages. They meet several of the aforementioned criteria, and readily offer themselves to the generation of high-quality influenza virus vaccines. The remainder of this review will focus on introducing the RNA replicon concept, summarizing previous work on replicon-based influenza vaccines, and presenting the future perspectives for this type of vaccine.

## 2. RNA Virus Vectors

Most RNA viruses are relatively simple and have small genomes (<15 kb). They are classified into positive- and negative-strand RNA viruses (single-stranded RNA genomes), or viruses with a double-stranded RNA genome. The genome of positive-strand RNA viruses (PSV) can directly function as mRNA when delivered into the host cell, and is therefore regarded as infectious. Translation of this RNA results in the generation of one or more polyproteins, which are proteolytically processed to produce a set of structural and non-structural proteins including the RNA-dependent RNA polymerase. This viral polymerase is essential for the transcription and replication of the viral RNA, which usually occurs in the infected cell cytosol independently of host nuclear functions (except for retroviruses).

Negative-strand RNA viruses (NSV) comprise viruses with segmented and non-segmented genomes. The genomic RNA of NSV is non-infectious (*i.e.,* transfection of *in vitro*-transcribed genomic RNA will not result in the generation of infectious virus). In contrast to PSV, the RNA genome of NSV has to be transcribed by a pre-existing viral RNA polymerase into positive-sense RNA. For this reason, the viral RNA polymerase protein complex forms an essential part of the virion, and remains associated with the viral RNA after infection to ensure initiation of transcription and replication. Although the majority of NSV replicate in the cytosol, some also require host nuclear functions (e.g., influenza viruses, borna disease virus). A further contrast to PSV is seen with the organization of non-segmented NSV genomes which are arranged in a modular, non-overlapping fashion, with each gene flanked by conserved transcription start and stop signals. A typical feature of non-segmented NSV is the gradient of transcription; that is, genes at the 3′ end of the genome are transcribed at higher levels than genes located further downstream. NSV with segmented RNA genomes, such as influenza virus, usually encode for one or two proteins per segment. Again contrasting with PSV, neither non-segmented nor segmented NSV genomes encode for polyproteins.

RNA viruses are open to genetic manipulation, and have been used as recombinant vaccine vectors for protection against a variety of pathogens [17]. In principle, these vectors represent live attenuated viruses expressing heterologous antigens. The advantage of such a vector concept can be realized with the following examples. Antigens originating from highly pathogenic viruses (e.g., ebolavirus), immunosuppressive or immunomodulatory viruses (e.g., HIV-1), or viruses that grow poorly in cell culture (e.g., hepatitis C virus) can be expressed by a well-characterized, attenuated vector virus that serves as the vaccine backbone. However, given the particularly high mutability of RNA viruses in general, propagation-competent RNA virus vectors may experience mutational changes leading to enhanced virulence. In addition, shedding of propagation-competent virus vectors from vaccinated individuals may be harmful to immunocompromised persons and the elderly. Indeed, it was as a result of such safety concerns that the Sabin polio vaccine was replaced by the safer Salk vaccine in Europe and North America, even though the Sabin vaccine had been successfully employed in the poliovirus eradication program for years [18,19]. Clearly, if one could employ a non-transmissible vector, this would prove particularly advantageous to vaccine design. Accordingly, the development of non-transmissible RNA replicons represents a major advance towards safe vector vaccines.

### 2.1. RNA replicons

RNA replicons are derived from either positive- or negative-strand RNA viruses, from which at least one gene encoding an essential structural protein has been deleted. RNA replicons can be regarded as “disabled” viruses unable to produce infectious progeny. Despite such gene deletions, the viral RNA is replicated and transcribed by the viral RNA polymerase. Any genetic information encoded by the replicon will be amplified many times, resulting in high levels of antigen expression. This property distinguishes RNA replicons from plasmid DNA-based vaccines, which rely on the initial levels of genetic information successfully delivered to the nucleus. The latter together with the characteristics of DNA replication can prove to be a major hurdle for advancing DNA vaccines. Certainly, vaccines based on DNA plasmids often contain regulatory sequences and antibiotic resistance genes. The potential integration of such sequences into the host genome by non-homologous recombination may represent an incalculable risk [20]. In contrast, replication/transcription of replicon RNA is strictly confined to the cytosol, and does not require any cDNA intermediates, nor is any recombination with or integration into the chromosomal DNA of the host required. Taken together, several safety issues are associated with DNA vaccines, which do not arise with the much more biosafe RNA-based vaccines.

### 2.2. RNA replicons and initiation of immune response development

Due to the fact that RNA replicons mediate intracellular antigen expression, they have the potential for efficiently stimulating both humoral and cellular immune responses, as would be the case with live attenuated virus vaccines. For the cellular responses, the vaccine antigens encoded by the RNA are subject to partial degradation by proteasomes in the cytosol. When this occurs in antigen-presenting cells (APC), the proteasomes can function as immunoproteasomes which are physically and functionally linked to TAP transporters (transporter associated with antigen presentation), promoting transport of the resulting peptides into the ER for loading on to MHC class I molecules. These are in turn transported to the APC surface for presenting the peptides to CD8+ T lymphocytes. Of the APC, dendritic cells (DC) are particularly well designated for triggering cellular immunity in this manner. A number of RNA viruses can directly infect DCs, but there are also viruses with a different tropism.

An alternative mode of antigen presentation relies on the induction of apoptosis in RNA replicon-infected cells (distinct from DCs). The antigen-loaded apoptotic vesicles can be efficiently taken up by DCs (or other antigen-presenting cells), which proteolytically degrade the antigens in acidified endocytic vesicles. These endocytic vesicles subsequently fuse with vesicles containing MHC class II molecules, the MHC class II compartment (MIIC). Stable association of the antigenic peptide with the MHC class II molecules leads to transportation of the complexes to the cell surface for presentation of the peptides to CD4+ T lymphocytes. In addition, DCs can present on their surfaces peptides derived from proteolytic degradation of internalized extracellular antigens via MHC class I molecules – a process known as cross-presentation [21]. With antigen presentation occuring in specialized lymphoid organs, DCs have to mature and migrate to these sites following uptake of antigen in the periphery. These processes are triggered by response of the DC to “danger” signals provided by pathogen-associated molecular patterns (PAMP), which include dsRNA or RNA containing 5′-triphosphate recognized by the Toll-like and RIG-I-like pattern recognition receptors (PRR), respectively [22]. Signalling through these receptors not only stimulates DC maturation, but also enhance antigen processing in general. Different DC subpopulations are certainly involved in these processes: Conventional or classical DC (cDC) are professional antigen presenting cells, while plasmacytoid DC (pDC) respond to particular “danger” signals to provide maturation factors acting upon cDC. While cDC can also respond to “danger” signals, the PRR of cDC and pDC can differ in this respect, and therefore the cells also differ in the PAMP they can recognize (see also the review on “DCs in innate and adaptive immunity responses against influenza viruses” by Summerfield & McCullough in this special issue of “Viruses”). The aforementioned response to RNA molecules is a strong property of pDC. Similar to live attenuated virus, RNA replicons can provide these signals and may therefore not be so reliant on costimulatory adjuvants as would be an inactivated protein-based vaccine. Thus, RNA replicons have high potential for efficiently stimulating both humoral and cellular adaptive immune defences. RNA replicons may also be manipulated with respect to their capacity to modulate type I interferon pathways, considering that the viruses from which the replicons were derived may themselves possess countermeasures against the hosts type I interferon system [23]. This modulatory activity may prove advantageous, but it may also be desirable to assure an efficient type I interferon response, considering the importance of this cytokine in DC maturation. RNA replicons offer themselves readily to such manipulation, to create entities with the desired immunomodulatory capacity. However, they usually do not express proteins that modulate or corrupt the adaptive immune system to the disadvantage of the host, as do certain large DNA viruses [24,25].

### 2.3. RNA replicons in current use for vaccine studies

Today, the most frequently used positive-strand RNA replicon vaccines are derived from (i) alphaviruses such as Sindbis virus, Semliki Forest virus (SFV), and Venezuelan Equine Encephalitis (VEE) virus [26,27], or (ii) flaviviruses such as Kunjin virus [28], West Nile virus [29,30], and tick-borne encephalitis virus [31]. Most human populations have no pre-existing immunity to these arthropod-borne viruses that would limit expression of the encoded heterologous antigen. Picornaviruses such as poliovirus have been also used as replicon vaccine vectors [32,33].

#### 2.3.1. Positive-strand RNA replicons for vaccine studies

**Figure 1.**
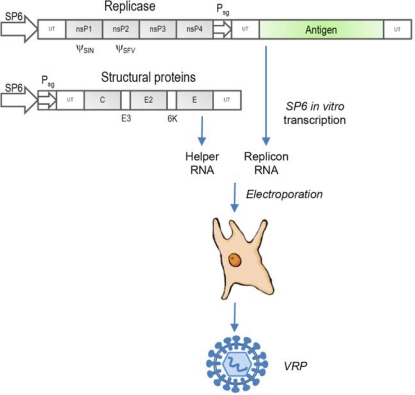
Generation of alphavirus replicon particles. The replicon RNA encodes for the foreign antigen and the viral non-structural proteins nsP1- nsP4 that form the active viral replicase. The helper RNA encodes for the three main structural proteins C, E2, and E1 and two minor proteins E3 and 6K. In order to minimize the risk of RNA recombination, the structural proteins are sometimes distributed on two separate helper RNAs (not shown). Following transfection of cells with the *in vitro* transcripts, a polyprotein is directly translated from the replicon RNA and proteolytically processed into the non-structural proteins that form the active RNA polymerase. This enzyme catalyzes the replication of both replicon and helper RNA as well as transcription from the subgenomic promoters (Psg). Following expression of the viral structural proteins, the replicon RNA (but not the helper RNA) is specifically packaged into viral replicon particles (VRP) that are released from the cell. UT, untranslated region. ψ_SIN_, ψ_SFV_, packaging signals of Sindbis virus and SFV, respectively.

Positive-strand RNA replicons may be generated in one of three different ways:

1. *Delivery of naked subgenomic RNA*. This approach requires *in vitro* transcription of RNA, which is then applied by intramuscular injection [32,34]. A drawback of this approach is the general liability of RNA potentially hampering such delivery *in vivo*.
2. *Delivery of DNA-layered RNA replicons*. This approach resembles a DNA vaccine approach. However, in contrast to conventional DNA vaccines, the DNA is transcribed in the nucleus into replicon RNA [35,36]. This RNA is exported from the nucleus and translated into a polyprotein leading to the synthesis of the viral RNA polymerase and amplification of the replicon RNA. As a consequence, high antigen expression levels can be achieved (in contrast to DNA vaccines). DNA-layered RNA replicons have the advantage that large-scale vaccine production under GMP conditions is feasible. A negative aspect is that, similar to DNA vaccines, recombination of vector DNA with chromosomal DNA may occur, and the other safety issues raised against the application of DNA vaccines enter into the argument.
3. *Generation of viral replicon particles (VRPs)*. VRPs based on alphaviruses are produced in mammalian cell lines that are transfected with a set of *in vitro* transcribed RNAs (Figure 1), including a replicon RNA that encodes the viral non-structural proteins along with the “carried” heterologous antigen [37,38] and a “helper” RNA that encodes the viral structural proteins. In some systems two separate helper RNAs are used to avoid RNA recombination and generation of propagation-competent viruses [39]. The helper RNAs contain the 5′ and 3′ ends of the virus and the viral subgenomic 26S promoter. Thus, in the presence of the subgenomic replicon, the helper RNAs are replicated and the structural proteins expressed. Due to this, virus replicon particles (VRPs) can form and be released into the cell culture supernatant. However, only subgenomic replicon RNA is packaged into VRPs, because the helper RNAs lack the cognate signal necessary for packaging. One disadvantage of the above is the necessity for transfection of multiple transcripts. In order to avoid such a procedure, packaging cell lines have been generated that constitutively express the encoded genes of the helper RNA. The helper RNAs are only translated upon transfection (electroporation) of replicon RNA, or following infection with previously generated VRPs. VRPs produced by such a method are infectious, and efficiently deliver their recombinant replicon RNA to any susceptible cell. However, normal cells as in a vaccinated host will not produce infectious progeny, because they cannot provide the viral structural proteins *in trans*, as do the specialized packaging cell lines designed specifically for VRP production. Thus, these RNA replicons are referred to as “non-transmissible”, “single-cycle”, or “propagation-incompetent” vectors. Sometimes, the denomination “replication-incompetent” vector is found in the literature. However, this designation is misleading considering that the ability of the replicons to replicate in the host cell without producing progeny is a very important property and advantage of these vaccine vectors.

#### 2.3.2. Negative-strand RNA replicons for vaccine studies

**Figure 2.**
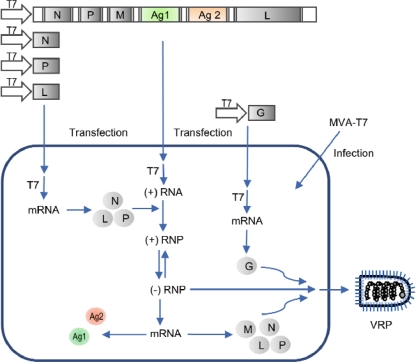
Generation of viral replicon particles based on non-segmented NSV. In this example, the replicon RNA encodes for the N, P and L proteins, which constitute the viral RNA polymerase complex, the matrix protein M, and two heterologous antigens Ag1 and Ag2. The replicon RNA lacks the gene encoding for the viral envelope glycoprotein G.

Negative-strand RNA replicons have been primarily generated with non-segmented NSV such as Sendai virus (SeV) or vesicular stomatitis virus (VSV). With the genomic RNA of these viruses being non-infectious *per se*, the viral RNA polymerase complex has to be present in the cell to initiate viral RNA replication and transcription. The first successful rescue of a recombinant non-segmented NSV solely from cDNA was performed with rabies virus [40]. The same principle can be applied to generate NSV-based replicons (Figure 2). At first, cells are infected with a recombinant vaccinia virus expressing the T7 phage RNA polymerase (MVA-T7). Subsequently, the cells are transfected with (i) plasmid encoding the viral RNA genome in positive (anti-genomic) orientation under control of the T7 promoter, and (ii) three helper plasmids driving the expression of the viral RNA polymerase complex under control of the T7 promoter. The viral RNA polymerase complex binds to the anti-genomic RNA, resulting in a positive-stranded ribonucleoprotein (RNP) complex, a natural intermediate in the viral life cycle, which can autonomously perform replication and transcription. If one wishes to generate replicons with deletions in one or more structural proteins, the respective component has to be provided by co-transfection of a suitable expression plasmid. SeV replicons have been constructed that have deletions in the envelope glycoprotein genes F and HN and/or the matrix protein M [41–46]. Similarly, VSV replicons lacking the envelope glycoprotein G have been produced [47,48].

Once the VRPs have been generated, they can be propagated on transgenic helper cell lines providing the deleted structural protein *in trans*. If this protein proves to be cytotoxic, a conditional expression system may be advantageous for generation of the helper cell line [41–43,49]. The deletion of genes encoding viral structural proteins has been shown to result in reduced cytotoxicity of the replicon when infecting non-helper cells [45]. Another consequence of gene deletions can be a diminution of replicon immunogenicity, in particular following deletion of the viral envelope proteins. When the replicon-infected cells did not express these antigens, the immune response directed against the virus envelope was low, and neutralizing antibodies were not induced [48]. This provided the advantage that the replicon vaccines could be repeatedly used in prime/booster vaccination protocols [36,48,50]. As with VRPs derived from enveloped positive-strand RNA viruses, VRP vaccines based on non-segmented NSV exploit the inherent receptor-binding and fusion activities of viral glycoproteins to efficiently deliver genetic information into cells. This makes the VRP system superior to any system for *in vivo* delivery of naked DNA or RNA.

Although NSV-based replicons have been less frequently used as vaccine vectors than replicons based on PSV, they reveal some features that make them particularly promising vectors. For example, non-segmented NSV have a module-like organization, which has high potential for hosting several recombinant antigens. Thanks to the typical transcriptional gradient of non-segmented NSV, different antigen expression levels can be achieved by using transcriptional units close to or remote from the 3′end of the genome. In contrast, PSV usually express large polyproteins that have to be proteolytically processed into individual viral components, which would be less favourable for the expression of multiple antigens. Moreover, RNA recombination seems to be more rare with non-segmented NSV [51,52] compared with PSV [53,54]. However, more research on this important aspect is needed to verify these preliminary observations.

### 2.4. RNA replicon vaccine studies

Influenza vaccines based on RNA replicons still represent experimental vaccines, which have not been evaluated in humans yet. Most studies have been performed in small animal systems, in particular in mice.

#### 2.4.1. Alphavirus replicons

Probably, the first experimental alphavirus replicon vaccine for protection against influenza was based on SFV. The replicon was engineered to express the influenza virus nucleoprotein (NP), and was applied to mice by intramuscular injection of either *in vitro* transcribed RNA or packaged VRPs [55]. Both versions of delivery resulted in strong humoral immune responses. In addition, immunization with minute amounts of VRPs (100 infectious units) induced efficient class I-restricted cytotoxic T-cell responses [56]. A DNA-layered SFV replicon vaccine has also been tested. Simultaneous intramuscular and intradermal immunization with replicons expressing NP and HA antigens of A/PR/8/1934 (H1N1) protected mice against a lethal influenza virus challenge [36]. In this study, the animals developed humoral and cellular immune responses at higher levels than mice receiving a conventional DNA vaccine vector. Vaccination with DNA-layered SFV replicons also induced long-lasting cytotoxic T-cell (CTL) memory that persisted in mice for over one year, and mucosal immunization resulted in the induction of secretory IgA in the respiratory tract. Development of immune responses directed against the vector itself did not inhibit boost responses by subsequent immunization with the same SFV replicon [57].

Some attempts have been made to improve the immunogenicity of HA antigen derived from a human H5N1 isolate by genetically fusing the antigen with the herpes simplex virus 1 VP22 protein then expressed by SFV VRPs [58]. Compared to replicons expressing authentic HA, higher IL-4 and IFN-gamma levels were induced in splenocytes with such replicons expressing VP22-HA.

A DNA-layered Sindbis virus replicon vaccine has also been used for expression of influenza virus HA antigen of A/PR/8/1934 (H1N1). In addition, replicon vaccines based on Venezuelan equine encephalitis (VEE) virus have been generated [39], in analogy to SFV and Sindbis virus replicons. These VEE VRPs were produced using a bipartite packaging system providing the VEE capsid protein and glycoproteins on separate RNAs *in trans*. This significantly improved the safety of the system, because regeneration of propagation-competent viruses was not detected. Two subcutaneous inoculations with VRPs expressing HA antigen of A/PR/8/1934 (H1N1) protected mice against intranasal challenge with the homologous influenza virus strain.

In contrast to previous studies evaluating alphavirus replicon vaccines in mice, a recent report provided evidence that alphaviral replicon particles expressing HA and NA antigens of A/Wyoming/3/2003 (H3N2) induced significant humoral and cellular immune responses in mice, rabbits and rhesus macaques with no adverse side effects [59]. However, the study lacks any challenge experiments that would provide information about the level of protection in non-rodent mammals. Although challenge experiments have been performed with other replicon vaccine studies, homologous virus was often used for infection of the immunised animals. Given the importance of influenza virus antigenic drift, it is evident that such studies are of limited value. Only a few studies have analysed the capacity of RNA replicon vaccines to stimulate cellular immune responses. Although cellular immunity is believed to play an important role in heterosubtypic protection, heterosubtypic challenge experiments have not been conducted yet.

#### 2.4.2. VSV replicons

VSV is a member of the family *Rhabdoviridae* and is endemic in parts of America. The virus is transmitted by insects to livestock such as cattle, horses, and pigs, causing a mild vesicular disease. Infections of man have been rarely reported, being described as being Flu-like [48]. In cell culture systems, VSV is characterized by a very broad cell tropism, short replication cycle (∼5 h), high titers (>10^9^ pfu/ml) and a pronounced cytopathogenicity. The VSV genome consists of about 11,200 nucleotides encoding five genes. Deletion of the G gene which encodes for the single envelope glycoprotein G resulted in a propagation-incompetent VSVΔG replicon that was initially used for pseudotyping experiments to characterize heterologous viral glycoproteins [47]. Later, VSVΔG replicons were used as experimental vaccines for protection against SARS [60,61], HIV-1 [61], hepatitis C virus [62], respiratory syncytial virus [63], and influenza viruses [48,50,64]. Roberts and coworkers showed that influenza virus HA antigen of A/WSN/33 (H1N1) was efficiently expressed by a VSVΔG replicon, being incorporated into the VSVΔG envelope [48]. Two intranasal inoculations with these VSVΔG-HA replicons protected mice from lethal challenge using the homologous influenza virus strain (A/WSN/33), and displayed no adverse effects. However, the VSVΔG-HA replicons were less efficient at inducing neutralizing antibodies than a propagation-competent vector expressing the same antigen. A subsequent study revealed that VSVΔG replicon vaccines are compromised for immunogenicity if applied to mucosal surfaces. Nevertheless, they show similar effectiveness as propagation-competent VSV vectors when delivered to mice via the intramuscular or intraperitoneal route [61]. Another study, again in mice, showed that VSVΔG replicon vaccines expressing the HA antigen of a human H5N1 isolate induced neutralizing antibodies against both the homologous strain and a number of other H5N1 strains [64]. Moreover, a single vaccine dose was sufficient to induce long-lasting protection (>7 months) against H5N1 challenge.

### 2.5. RNA replicon vaccines for protection of poultry

In many developed countries general vaccination of poultry against highly pathogenic avian influenza viruses (HPAIV) is not allowed, a policy based in principal on two arguments. Firstly, the currently available inactivated influenza virus vaccines may protect poultry in the sense that they do not show signs of diseases. However, inactivated vaccines are inefficient in providing sterilizing immunity, such that vaccinated animals may shed significant amounts of virus. Such vaccines may even expedite viral antigenic drift by selection of escape mutants [65]. Secondly, whole inactivated influenza virus vaccines do not facilitate discrimination between infected and vaccinated animals (DIVA). The use of inactivated vaccines may therefore lead to undetected spread of AIV and undesired prohibition of poultry trading. Moreover, to meet the requirements of modern poultry husbandry, AIV vaccines must allow mass application at low costs. Although live attenuated influenza viruses may provide superior protection, they are not acceptable due to the potential of permitting reassortant formation and promoting antigenic drift [66]. Thus, in many aspects the demands on influenza vaccines for use in poultry are higher compared to influenza vaccines for human use. Nevertheless, given the zoonotic potential of avian influenza viruses, vaccination of poultry would be important from both an economical and public health point of view.

Experimental RNA replicon vaccines have demonstrated potential as novel marker vaccines for poultry. Importantly, alphaviruses such as VEE or rhabdoviruses such as VSV are not natural avian pathogens, thus excluding that birds would possess any pre-existing immunity against the viral vectors. Immunization with a single dose of VEE VRPs expressing the HA antigen of an H5N1 isolate resulted in complete protection of chickens against lethal challenge with the homologous virus [67].

The HA protein is certainly a most important influenza virus antigen for inducing neutralizing antibodies. Accordingly, in most studies this antigen has been used for immunization. Nevertheless, the role of the NA antigen in protection against avian influenza viruses has also been studied. Sylte and co-workers immunized chickens with VEE VRPs driving the expression of the NA antigen from A/pheasant/Maryland/4457/93 [68]. They found that chickens receiving two vaccine doses were partially protected from challenge with the parental HPAIV, whereas no protection was achieved against challenge with a genetically distant influenza virus. This and similar findings by others [69,70] suggest that the NA antigen does not induce full protection on its own, but may contribute to immunity against influenza viruses.

VSVΔG replicons encoding either HA or NP antigen of an HPAIV (H7N1) strain were also evaluated as vaccines for chickens [50]. HA was efficiently expressed by this replicon vector, but did not substitute for the deleted VSV G protein functions, although HA also has receptor-binding and fusion activities. Chickens receiving a prime/booster immunization regime with this replicon were protected against lethal challenge with heterologous HPAIV (H7N1) influenza virus, even though the HA of the challenge virus differed from the primary amino acid sequence of the vaccine antigen by 8%. In addition, shedding of challenge virus from the vaccinated birds was only transient and significantly reduced. Moreover, it was possible to discriminate between vaccinated and infected animals by a simple ELISA assay for detection of antibodies directed to NA. The additional application of a replicon expressing NP antigen was also tested, but no additional beneficial effect on virus secretion was observed. This is not surprising given that the replication of HPAIV in chicken is likely to be too fast to allow an efficient cellular immune defence to be mounted.

### 2.6. Immunological correlates of protection

The immune correlates of protection against influenza virus infection and disease have been only partially addressed over recent years, leaving an important gap in our current knowledge on what is required [71]. An intriguing study showed that a conventional DNA vaccine vector expressing HA antigen induced neutralizing antibodies in mice, which correlated with increased IgG1 levels [72]. In contrast, VEE VRPs expressing HA antigen did not induce IgG1 or neutralizing antibodies. Rather, antibodies of the IgG2a isotype were induced, but these did correlate with virus clearance and increased protection against lethal influenza challenge. These findings certainly argue for the development of vaccine regimens that stimulate both antibody isotypes, but one also has to consider the immunological differences between rodents and humans when attempting to elaborate on these results. Clearly, more information is required on the immune response of mammals other than mice, and also the relative roles played by humoral and cytotoxic immune defences.

Another aspect of immune correlates which is also particularly poorly studied is the importance of local immunity in resistance to influenza virus induced disease. Being a respiratory infection, one would consider that mucosal immunity would be of prime importance in the ability of the host to resist the virus and the disease it can induce. Influenza viruses replicate predominantly in the epithelial cells of the respiratory tract. Therefore, mucosal antibodies (IgA) are believed to represent an important line of defence. Although antigen processing across mucosal surfaces is the natural route by which mucosal immunity is generated, VEE VRPs have been reported to induce mucosal immunity even when applied parenterally [73]. Both mucosal and systemic immunity was induced when VRPs not expressing any influenza virus antigen were co-inoculated with inactivated influenza virions, suggesting that the particles possess intrinsic adjuvant activity. This activity basically relied on viral RNA replication, because UV-treated VRPs showed no such activity [73]. Induction of type 1 interferon (IFN) was not essential for the adjuvant effect, but appeared to be important for VRP-induced immunity – HA-VRP vaccination of IFN receptor knock-out mice failed to induce significant levels of influenza virus-specific mucosal IgA antibodies [74].

## 3. Resume and future perspectives

Presently, inactivated subunit vaccines are commonly used for immunoprophylaxis of influenza virus infection. While clear advantage of subunit vaccines is their safety, their usage has come with a number of drawbacks including low efficacy (in particular in the elderly), low cross-protective immunity, and short-term immunity (Table 1). In principle, live attenuated, cold-adapted influenza virus vaccines should be more efficient at inducing both humoral and cellular immune responses, as well as providing long-lasting protection against antigenic drift or even heterosubtypic influenza viruses [75]. Another advantage is that cold-adapted influenza viruses can be applied intranasally, and thus induce mucosal immunity. However, RNA viruses are particularly prone to mutational changes, for which reason it cannot be completely excluded that virulent revertants will evolve when employing live, cold-adapted influenza virus vaccines. Moreover, due to the segmented character of their RNA genome, such live vaccines may form reassortants with field strains, thus excluding their use in times of influenza virus epidemics and pandemics – the times when vaccination is most needed. Finally, live attenuated influenza virus vaccines will not be accepted in veterinary medicine, because these do not facilitate the distinction between infected and vaccinated animals by serological assay.

RNA replicons may show a way out of the above dilemma. They combine the safety of inactivated vaccines with the superior immunogenicity of live, attenuated vaccines (Table 1). Nevertheless, the development of RNA replicon vaccines against influenza is still at an early stage relative to the more established subunit vaccines. There are a number of characteristics which still need to be defined in terms of their delivery to the host, their efficacy at inducing immune defences when applied as a single shot or with booster immunization, the correlates of immunity induced relative to protection, and the possibility to apply by the intranasal route. With such information, one can meet the requirements of high quality influenza virus vaccines.

### 3.1. RNA replicon vaccines for multiple antigen expression

Most studies on RNA replicon vaccines so far were based on vectors driving single expression of HA, the major influenza virus antigen particularly subject to antigenic drift. In order to approach the efficacy of live attenuated vaccines with respect to cross-protection against drift and heterosubtypic viruses, RNA replicons have to be generated that mediate the expression of multiple influenza antigens. One particular element therein would be the highly conserved internal virus antigens, which contain dominant T cell epitopes [76,77]. The cellular immune response triggered by these antigens is expected to mediate enhanced virus clearance and to reduce both morbidity and mortality [11,78]. While expression of multiple antigens by individual PSV-based replicons may prove rather limited, a vaccine could contain a cocktail of replicons each expressing a different influenza virus antigen. Of course, RNA replicons based on non-segmented NSV are adaptable vectors for simultaneous expression of multiple antigens. The module-like organization of their RNA genome may allow expression of two or three different antigens from a single replicon. Moreover, the combination of two such NSV replicons would provide a more potent multiple antigen vaccine.

### 3.2. RNA replicon vaccines for mucosal immunizations

The respiratory tract is lined by epithelial cells organized in a polar fashion [79]. Influenza viruses efficiently infect polarized epithelial cells via the apical membrane. This contrasts with entry of many arthropod-borne viruses, which is restricted to the basolateral membrane [80,81], in line with their transmission by insect bites or injections. The property of the latter may explain why the intramuscular or intradermal route is normally used for immunization with RNA replicons that are derived from arthropod-borne viruses. Although it has been reported that alphavirus replicons can induce mucosal IgA antibodies to some extent [57,73], replicon vaccines that can be applied directly to mucosal surfaces are highly desirable, because this would mimic the natural infection route of influenza viruses. Enveloped viruses may be re-targeted to specific cell subsets by acquisition of a foreign envelope-inserted protein, a process known as “pseudotyping“. Unfortunately, pseudotyping of alphaviruses has not been achieved so far. In contrast, VSV is amenable to pseudotyping, readily incorporating foreign viral glycoproteins in a more or less non-selective manner [82], particularly when the VSV G glycoprotein is absent [47]. Interestingly, pseudotyping of VSVΔG replicons with the influenza C virus glycoprotein resulted in efficient apical infection of polarized epithelial cells [49]. These pseudotyped VSV replicons can now be used to test the hypothesis that apical infection of respiratory epithelial cells induces stronger mucosal immune responses compared with replicons applied parenterally. An attractive alternative to VSV pseudotypes for mucosal immunization are RNA replicons derived from non-segmented NSV showing a natural tropism for the respiratory tract. Sendai virus replicons would hold promise in this regard.

### 3.3. RNA replicon vaccines for the future

The inability of RNA replicons to produce infectious progeny is a clear plus with respect to biosafety. However, it limits the dissemination of antigen and thus the number of dendritic cells having access to the antigen. This lack in antigen dissemination may be overcome by expression of recombinant soluble antigens [83,84] that can be secreted by the cell. Alternatively, expression of costimulatory cytokines and chemokines by the replicon, which are released by the cell, may enhance recruitment of dendritic cells to the site of vaccine delivery [85]. Of course, the “ideal” RNA replicon vectors in terms of vaccination efficacy should be a replicon which specifically targets dendritic cells [86,87]. Such replicons must not be cytotoxic to allow dendritic cells to perform their immunological roles in processing the vaccine antigens, maturing and migrating to lymph nodes for activation of the adaptive immune responses.

This review has referenced our current knowledge on RNA replicon vaccines, which have been mostly evaluated in mice to date. In future, it will be necessary to demonstrate that RNA replicons also provide protection of larger non-rodent mammals such as pigs and ferrets. Protection of vaccinated animals has tended to analyse challenge with homologous virus, but this should now be elaborated to include challenge analyses using heterologous virus. Such challenge infections are clearly needed to test the quality and value of a modern influenza virus vaccine. The induction of cellular immune responses providing long-lasting immunity against heterosubtypic influenza viruses will certainly be an important aspect in future vaccine developments, an element which can be offered by the RNA replicon vaccine.

The rapid availability of any influenza virus vaccine is a crucial aspect, particularly in face of an influenza virus pandemic as we recently experienced when the new H1N1 virus emerged in 2009. Production of alphavirus replicon vaccines by transfecting approved cell lines with naked RNA or cDNA may be straightforward, because these procedures can be easily performed under conditions of Good Manufacturing Practice (GMP). However, the use of specialised packaging cell lines to generate VRP holds considerable promise for generating effective vaccines, because such VRPs are based on the inherent viral delivery systems for their own genetic material. A crucial aspect here is the generation of approved transgenic helper or packaging cell lines for propagation of RNA replicons. These cell lines must allow high yield propagation of RNA replicons, and comply with the highest biosafety standards. In particular, the regeneration of propagation-competent viruses by RNA recombination must be excluded. These problems are likely to be solved in the very near future so that VRPs can be produced in approved mammalian cell lines akin to other biologicals.

## Figures and Tables

**Table 1.** Comparison of RNA replicon vaccines with classical influenza virus vaccine concepts.

**Parameter**	**Inactivated subunit vaccine**	**Live attenuated vaccine**	**RNA replicon vaccine**
Safety	High	Risk of revertants, reassortants	High
Induction of humoral immunity	Yes	Yes	Yes
Induction of cellular immunity	Low	Yes	Yes
Mucosal Immunity	Low	Yes	Not yet tested
Booster Immunisation	May be required (dependent on the vaccine)	No	Likely to be required (further work needed)
Immune memory	Short-term	Long-term	Long-term
Protection against drift virus	Low	Yes	Yes
Heterosubtypic protection	No	Yes	Not known
Shedding of vaccine virus	No	Yes	No
Application route	Parenteral (Intranasal possible, but there are safety queries*)	Parenteral or Intranasal*	Parenteral (Intranasal under investigation*)
Adjuvants	Not used**	Not usually required	Not usually required
Antigens	HA, NA	Multiple	Maximum number yet to be determined
Availability	Low	High	High

* Intranasal vaccination with influenza virus vaccines has met with a series of hurdles, due to reports in the literature of problems when vaccinating humans by this route. The crux of the problem may well lie with the adjuvant, but there is now the query as to how the vaccine may also be involved, particularly but not only when applied with an adjuvant. A replicon vaccine may prove to be more readily acceptable for intranasal clinical trials, but safety studies are likely to be required prior to intranasal testing in humans.

** In contrast to many other inactivated vaccines, seasonal influenza virus vaccines are commonly used without adjuvants.
